# Should age matter in COVID-19 triage? A deliberative study

**DOI:** 10.1136/medethics-2020-107071

**Published:** 2021-03-09

**Authors:** Margot N I Kuylen, Scott Y Kim, Alexander Ruck Keene, Gareth S Owen

**Affiliations:** 1 Department of Psychological Medicine, King's College London, London, UK; 2 Department of Bioethics, National Institutes of Health, Bethesda, Maryland, USA; 3 Center for Bioethics and Social Sciences, University of Michigan, Ann Arbor, Michigan, USA; 4 39 Essex Chambers, London, UK; 5 Dickson Poon School of Law, King's College London, London, UK

**Keywords:** covid-19, resource allocation, ethics

## Abstract

The COVID-19 pandemic put a large burden on many healthcare systems, causing fears about resource scarcity and triage. Several COVID-19 guidelines included age as an explicit factor and practices of both triage and ‘anticipatory triage’ likely limited access to hospital care for elderly patients, especially those in care homes. To ensure the legitimacy of triage guidelines, which affect the public, it is important to engage the public’s moral intuitions. Our study aimed to explore general public views in the UK on the role of age, and related factors like frailty and quality of life, in triage during the COVID-19 pandemic. We held online deliberative workshops with members of the general public (n=22). Participants were guided through a deliberative process to maximise eliciting informed and considered preferences. Participants generally accepted the need for triage but strongly rejected ‘fair innings’ and ‘life projects’ principles as justifications for age-based allocation. They were also wary of the ‘maximise life-years’ principle, preferring to maximise the number of lives rather than life years saved. Although they did not arrive at a unified recommendation of one principle, a concern for three core principles and values eventually emerged: equality, efficiency and vulnerability. While these remain difficult to fully respect at once, they captured a considered, multifaceted consensus: utilitarian considerations of efficiency should be tempered with a concern for equality and vulnerability. This ‘triad’ of ethical principles may be a useful structure to guide ethical deliberation as societies negotiate the conflicting ethical demands of triage.

## Introduction

The first wave of the COVID-19 pandemic put a large burden on many healthcare systems. Fears arose that demand for resources would exceed supply, necessitating triage in critical care, for example, when allocating intensive care unit (ICU) beds. The role of age in resource allocation was an especially salient issue given the proclivity of SARS-CoV-2 to cause excess mortality in older groups. Several COVID-19 triage guidelines included age as an explicit factor,^1–4^ and practices of both triage and ‘anticipatory triage’ likely limited access to hospital care for elderly patients, especially those in care homes.^5–8^ This raised ethical and societal questions about the role of age in triage decision making.^9–11^

In medical ethics literature, different principles for resource allocation exist. Following a scoping review, we identified four that have explicit implications for the use of age as a deciding factor in triage:(1) the ‘fair innings’ principle, (2) the ‘life projects’ principle, (3) the ‘egalitarian principle’ and (4) the ‘maximise life years’ principle. (1) The ‘fair innings’ principle prioritises younger over older people so that younger people also get the chance to reach later life stages.^12^ (2) The ‘life projects’ principle prioritises young to middle-aged people so that everyone gets the chance to complete their life projects (eg, raising children and making a career).^13^ (3) The egalitarian principle calls for equal treatment of all and does not permit discrimination on the basis of age, meaning we must take a ‘lottery’ or ‘first come, first served’ approach.^14 15^ (4) Finally, the ‘maximise life years’ principle, a utilitarian approach, permits indirect discrimination on the basis of age insofar as this maximises the amount of life years saved.^16^

These principles have conflicting implications. Our study aimed to explore general public views on the role of age in triage decision making during the COVID-19 pandemic. Specifically, we wanted to understand attitudes to the aforementioned four allocation principles, as well as on related factors such as quality of life and frailty. We also sought to understand, and elicit, participants’ considered recommendations on triage, with a view to developing ethical guidelines that are sensitive to public thinking.

## Methods

We held deliberative workshops with members of the general public following the general method of deliberative democracy,^17–19^ in collaboration with UK market research company Ipsos MORI, which has expertise in deliberative workshops. We requested them to recruit 25 participants from South East London, so as to inform clinical ethics forums in hospitals associated with King’s College London. Participants were guided through a deliberative process so they could arrive at an informed and considered opinion on topics that may have been new or unfamiliar to them. Four workshops, each lasting 2 hours, took place during 3 weeks across August and September 2020, in a particular social window between the first and second wave of COVID-19. This was an opportunity for participants to discuss the complex ethical questions on triage in a context in which its importance was pertinent. Three participants dropped out before the first session for personal reasons. Nineteen participants took part in all four sessions; the three remaining participants each took part in three out of four sessions.

Deliberative democracy offers medical ethics a promising way to consult public preferences while ensuring these are adequately informed and considered. The sessions met the three standards for deliberation set out by Blacksher *et al*.^20^ First, sessions included informative presentations to provide ‘balanced, factual information that improves participant’s knowledge of the issue’. Second, we ensured ‘the inclusion of diverse perspectives’ through strategic sampling: participants reflected the demographics of the demographically diverse boroughs of Lambeth and Southwark (see table 1 for sample characteristics). We made particular effort to include participants over 60 years. Third, participants were given ‘the opportunity to reflect on and discuss freely a wide spectrum of viewpoints and to challenge and test competing moral claims’: the sessions included plenary discussions and discussions in smaller breakout groups, which were facilitated by experienced qualitative research staff from Ipsos MORI. Facilitation was non-directive and neutral with respect to content but active in promotion of an engaged, inclusive process among participants.

**Table 1:** Participant demographics

Gender	Male	12
	Female	13
Age (years)	20–29	4
	30–39	5
	40–49	3
	50–64	5
	65+	8
Ethnicity	White	12
	Black	7
	Asian	3
	Mixed	3
Socioeconomic grade (based on chief occupation of head of household)	B (higher and intermediate managerial, administrative, professional occupations)	4
	C1 (supervisory, clerical and junior managerial, administrative, professional occupations)	9
	C2 (skilled manual occupations)	6
	D (semiskilled and unskilled manual occupations)	2
	E (unemployed and lowest grade occupations)	4
Working status	In full-time employment	8
	In part-time employment	6
	Currently not in paid employment	4
	Retired	7
Highest level of education	No qualifications	3
	GCSE Level or equivalent (usually age 16 years)	4
	A-Level or equivalent/vocational qualifications (usually age 18 years)	5
	College/university degree level or equivalent	12
	Postgraduate degree level	1

GCSE, General Certificates of Secondary Education.

The research team (GO, MNIK, ARK) observed sessions and held discussion with the facilitators between workshops. The sessions were transcribed by professional note takers, and transcriptions were thematically analysed in two stages. First, general themes were identified in the raw data by Ipsos MORI and the research team and summarised in the report. In a second step, the research team analysed the raw data again with particular focus on the ethical reasoning underlying discussions.

Ahead of the study, we worked with Ipsos MORI to develop a detailed but accessible discussion guide for the workshops and survey questions to be answered by participants after each session. We also developed information materials to present to participants: a presentation on how resource allocation and treatment escalation works in England’s National Health Service, an overview of relevant data on how COVID-19 affects the elderly, video presentations spelling out the four allocation principles, materials explaining the concepts of frailty and quality of life and case vignettes showing how triage dilemmas may arise. These materials and further details of the methods are reported elsewhere.^21^

During session 1, the information materials were presented to participants, and initial reactions to the four principles were briefly explored in breakout groups. During session 2, case study examples were discussed in breakout groups to examine the practical implications of the respective principles. During session 3, participants were introduced to the notions of frailty and quality of life and explored these in breakout groups through one further hypothetical triage dilemma. Participants also deliberated further on the four principles and were asked to spell out their concerns about them. During session 4, participants were asked to formulate final recommendations and caveats in breakout groups. They also discussed how recommendations should be implemented and communicated to the public.

Given pandemic safety measures, the workshops were conducted online on Zoom. This was a relatively novel approach to deliberative democracy. Benefits of this approach were that participants felt more comfortable expressing opinions about sensitive subjects, carers or family members could more easily support older or vulnerable participants to contribute to the deliberations, and there was more time between sessions for reflection than with face-to-face sessions, which usually take place within 1 day. Downsides were that some participants experienced minor technical difficulties.

All participants gave informed consent before taking part.

## Findings

### ‘Fair innings’ and ‘life projects’ principles

The ‘fair innings’ and ‘life projects’ principle were strongly rejected from the outset and throughout the deliberative process. Participants found the ‘fair innings’ principle arbitrary and unnuanced, as well as unfair. They felt that age alone does not provide sufficient information about someone’s medical condition and that the lives of older people are important too: ‘We should get all equal treatment, young or old, we’re all the same’. Some participants also mentioned the contributions of the elderly to society, stating that ‘older people have just as much to give to society as younger people do’. The ‘life projects’ principle was equally firmly rejected, on the basis that it was normalising, favouring existing societal norms that not everyone meets: ‘It’s very discriminatory and not right. There are late developers. There are people who bloom later or earlier in life’. It was also emphasised that retirement was a time in which, after a life of work, people are finally free to start and pursue their life projects: ‘When you get older, that’s when you want to start projects. […] There are a lot of people almost having second lives doing all the things they couldn’t do previously’. Dismissing this period, therefore, seemed counterintuitive.

### Egalitarian principle

The egalitarian principle was accepted, though a number of concerns about it were raised throughout the study. Initially, this principle was received as the most straightforward and fairest principle, but as discussion progressed, worries emerged about its practical application. First of all, participants rejected a randomised ‘lottery’ approach, preferring a ‘first come, first served’ version of this principle: ‘lottery doesn’t feel like a good system when it’s people lives. It’s inappropriate’. But even the latter approach raised concerns. Participants were mostly worried about hidden inequalities, stating this approach would not redress, and even risk reinforcing, existing inequalities (eg, people with better access to the hospital may get there sooner). One participant said that ‘first come, first served isn’t egalitarian and you have the socio-economic challenges because, if you are in a particular class, you’re in a better position to be able to take care of yourself and get to the doctors first’. There were further concerns that a ‘first come, first served’ approach would waste valuable resources, when patients with a worse prognosis happen to arrive earlier. Finally, some participants felt uneasy that, on this approach, resources would not necessarily go to those who need them most: ‘On the face of it, it looks good, but I think means that those that come in later who are in greater need haven’t got access’. A few participants remained in favour of an egalitarian approach, though all accepted that, if a patient’s prognosis is extremely poor, they should not be escalated for treatment: ‘if you were following the egalitarian principle but you have someone in front of you who the evidence would suggest is highly unlikely to survive treatment and you’ve got someone who is highly likely to survive, as unfair as it may seem, it feels like it would be an important consideration […] I’m only thinking about extreme cases where you’ve got someone who is extremely frail and therefore extremely unlikely to survive’.

### ‘Maximise life years’ principle

When the ‘maximise life years’ principle was introduced, immediate concerns were raised about the accuracy of medical judgments about life expectancy: ‘Nobody knows how long anybody is going to live for. There are some assumptions, even if you’ve got two people in front of you, one who is 40 and one who is 60’. Furthermore, in discussing this principle, participants spontaneously distinguished survival chance from life expectancy in the deliberations and strongly favoured the former. They supported maximising the number of *lives* saved, rather than the amount of *life years* saved: ‘There’s a logic in maximum number of lives you save irrespective of the number of life years they have’. The underlying reasoning seemed to be that every life is of equal value: a majority of participants agreed that ‘a life is a life’.

It was thus widely felt that a patient’s immediate medical condition was a very important factor in triage, insofar as this informed their chances of survival. In this context, participants recognised frailty as a key factor: though it was not initially understood as a medical term, it was eventually accepted as a relevant prognostic variable for predicting survival chances.

Some participants questioned the survival chance-based approach, though. For example, a small number of participants expressed concern about the disproportionate effects it could have on groups that may be more vulnerable to COVID-19: ‘By virtue of prioritising survival of the fittest, it will discriminate and people are uncomfortable with this because it means older people will be less likely to be escalated, people in wheelchairs, people in BAME communities’. Another more widespread worry was that this approach failed to allocate resources in accordance with need. These concerns led some participants to formulate a new, vulnerability-based allocation principle, which is discussed further below.

### Quality of life

The notion of quality of life was initially treated with suspicion, seen as inviting unconscious bias and too subjective: ‘I don’t know if professionals can really confirm how somebody’s well-being is’. Throughout the study, it was increasingly accepted, though mostly as a secondary factor when patients’ medical conditions are highly similar, in which case those with a higher quality of life would be prioritised. Caveats were that it should only be applied in extreme cases and that quality of life assessments should, where possible, involve ‘input of the person, their family, carers and that kind of stuff’ to avoid biased assessments.

However, one participant said those with a *lower* quality of life should be prioritised, so that their quality of life may be improved. Some also noted that quality of life may be strongly influenced by socioeconomic factors, indicating a danger of exacerbating existing inequalities: ‘I do worry with quality of life, the more money you have, the better quality of life you tend to have […] your health is defined by your class and how much money you have’.

### Vulnerability

Throughout the study, concerns were expressed about *vulnerability,* especially in reaction to the utilitarian approach. In these discussions, participants struggled to formulate an additional allocation principle. This had two aspects, though these were not always clearly differentiated. One aspect concerned vulnerable *groups* (eg, age, disability or ethnic groups) who may be disproportionately affected by the virus itself or the social response to it (eg, unconscious bias). One participant said: ‘we know it affects the elderly at higher rates than the youth. […] It makes the most sense to prioritise the elderly over the young, just on the basis of the percentages of old people vs young people dying. Young people are more likely to survive’. There was, however, some disagreement over whether positive action for these groups should indeed be taken to mitigate the vulnerability or whether this was itself a form of discrimination.

The other aspect concerned *individuals* in need (eg, those presenting to hospital as sicker) and whether a humane principle was to prioritise those in greatest medical need: ‘The more help somebody needs, the more they should get’. Some suggested to prioritise those *least* likely to survive: ‘I think the most vulnerable should be prioritised. […] If you think you can save them, then prioritise them’. Reasons given for such an approach were that ‘the true measure of any society is how it treats its most vulnerable members’. But, again, it was accepted that if treatment was unlikely to succeed, patients should not be escalated: ‘you give the resources to the people that most need it, in my opinion, up until the point where the giving of resources is next to useless, where it’s ascertained that they will die anyway’.

Other participants rejected this need-based approach altogether, out of a concern for efficiency: ‘Does that mean, if those people are most likely to die, you’re directing your resources at people who are weaker? So resources could be going to a group who stand the least chance of surviving? That doesn’t feel like a great use of resources’.

### Implementation

During the final workshop, participants were asked how their recommendations should be implemented. We found strong support for discretion (applying recommendations as guidance rather than a mandatory policy), and participants felt groups of doctors, not individuals, should make decisions as this could reduce burden and bias. Thus, guidelines should not be binding but instead guide expert deliberation, and this deliberation is ideally executed by teams rather than individuals, so that different perspectives can be considered.

## Discussion

In summary, we observed a strong rejection of the two explicitly age-based principles; a tolerance for an egalitarian ‘first come, first served’ principle, though with doubts about sufficiency; wide support for a newly formulated approach based on survival chances, with some consideration of frailty and quality of life; concerns about group vulnerability and individual need; and a preference for discretion and deliberation in triage decision making.

These findings raise important questions regarding existing guidelines and expert recommendations, when and where they do not align with them. Fallucchi *et al* 22 have observed similar public intuitions, which digress from US triage guidelines, but conclude that the public requires more education. We found, however, that these public moral intuitions persist even after a robust process of reflection and deliberation. We think this warrants serious consideration of public preferences.

A first preference deserving serious consideration is the stark rejection of direct discrimination on the basis of age, as well as the use of randomised ‘lottery’ approaches, both of which have been observed in similar studies.^22 23^

A second focal point is the preference for survival chance over life expectancy, which also has been observed elsewhere.^19 22^ Savulescu *et al* 24 have criticised the UK’s NICE guidelines on resource allocation during COVID-19^25^ for including considerations of survival chance but not life expectancy. The NICE guidelines reject the latter as it results in indirect discrimination on the basis of age. According to Savulescu *et al*, however, the guidelines *already* tolerate indirect discrimination since basing triage on survival chance will *also* disproportionally affect the elderly. The authors thus assume both factors operate on the same logic. However, we suspect our participants may have highlighted an ethically relevant distinction between survival chance and life expectancy. In fact, there are at least two ways in which these factors may be different. First, considering life expectancy in triage seems closer to *direct* age-based discrimination. While survival chance is closely linked to age specifically in the context of COVID-19, life expectancy has a closer (indeed almost conceptual) link to age: to be older simply *is* to be closer to death. A similar distinction between survival chance and life expectancy has been made by Mello *et al*,^26^ who argue that only the latter results in *disability*-based discrimination. Second, a live saved and a life year saved seem to produce a different kind of value: a life saved is a categorical outcome, whereas a life year saved is a scalar outcome. This conceptual difference seems ethically relevant because most participants considered *any* life saved of inherent value, regardless of its predicted length: it is ‘about saving as many people as possible, even if they have a shorter life’. On this logic, saving *more of a life* does not produce additional value.

A third finding deserving of consideration is the concern about vulnerability. The core values of equality and efficiency, and the question of how to balance both, are central to discussions about resource allocation. During our study, however, a third relevant principle spontaneously emerged from the discussions: vulnerability. Though this notion was not unpacked in much detail during the deliberations, it alludes to values of antidiscrimination and protection, in line with emerging debates in the literature.^27 28^

How can these public intuitions be incorporated into triage decisions? Participants generally accepted the need for triage but did not arrive at a unified recommendation of one principle; indeed, in the final survey, recommendations included a mixture of principles and factors. However, a concern for three core principles and values emerged. As mentioned, deliberation resulted in the formulation of three broad, but distinguishable, allocation principles: an egalitarian ‘first come, first served’ principle, a utilitarian principle (but based mainly on survival chance and frailty) and a ‘vulnerability’ principle. The underlying core values of each of these principles could be described as equality, efficiency and vulnerability, respectively. In other words, a ‘triad’ of ethical values emerged. While these remain very hard to fully respect at once, they captured a considered, multifaceted consensus. All three principles were embedded in caveats and raised their own set of concerns. Notably, for each principle, these caveats and concerns can be linked back to the two *other* values of the triad:

- The egalitarian ‘equality’ principle raised concerns about efficiency and vulnerability: if treatment was likely futile, it was agreed that patients should forgo it (efficiency concern); participants worried strongly about hidden inequalities (vulnerability concern).
- The ‘efficiency’ principle raised concerns about equality and vulnerability: most agreed that if there was a ‘close call’ between patients, an egalitarian approach should be adopted instead (equality concern); some worried about groups more vulnerable to COVID-19 and about individuals with greater clinical need (vulnerability concerns).
- The ‘vulnerability’ principle raised concerns about equality and efficiency: many participants resisted the notion of positive discrimination for vulnerable groups (equality concern); many also worried that scarce resources would be ‘wasted’ on vulnerable individuals as they may not survive or take up more time in ICU (efficiency concerns).

We are hopeful, therefore, that this ‘triad’ of ethical principles may be a useful structure to guide ethical deliberation as societies negotiate the conflicting ethical demands of triage.

This links to our finding that participants favoured discretion and group deliberation in triage decisions. In light of this, the triad may offer a useful framework, as it does not prescribe one single principle but rather a balancing exercise among three core values, ideally performed by a team of deliberators. In sum, rather than inviting moral paralysis, we hope this triad could guide fruitful case discussion for doctors, reduce moral distress and give them more confidence that the triage decisions they arrive at have public acceptability.

## Strengths and limitations

### Strengths

We achieved a purposeful sample, there was a high level of participant engagement, participants showed they could think through complex ethical topics, a triad consensus emerged from a very diverse South-East London group, indicating a degree of robustness and there was the ecological validity of doing this study in the social window in between two COVID-19 waves.

### Limitations

The South-East London sample may not generalise to other areas, findings may not generalise to other triage contexts (eg, pandemics effecting children) and some elements, for example, vulnerability, remained underexplored, indicating a need for further research.

## Conclusion

To ensure the legitimacy of triage guidelines, which affect the public, it is important to engage the public’s moral intuitions, as they do not always align with expert recommendations. Guiding the public through a process of deliberation ensures that public intuitions do not stem from ignorance or misunderstanding but rather express genuine and considered preferences. We found that (widespread) utilitarian considerations of efficiency should be tempered with a concern for equality and vulnerability.

## Data Availability

No data are available.
